# SubFoveal Choroidal Imaging in High Myopic Nepalese Cohort

**DOI:** 10.1155/2023/2209496

**Published:** 2023-05-11

**Authors:** Parash Gyawali, Ashutosh Jnawali, Anish Kharal, Manish Subedi, Sandeep Kandel, Prajjol Raj Puri, Nabin Paudel

**Affiliations:** ^1^B. P. Koirala Lions Center for Ophthalmic Studies, Institute of Medicine, Tribhuvan University, Kirtipur, Nepal; ^2^College of Optometry, The University of Houston, Houston, Texas, USA; ^3^Tilganga Institute of Ophthalmology, Kathmandu, Nepal; ^4^Centre for Eye Research Ireland, Technological University Dublin, Dublin, Ireland

## Abstract

**Purpose:**

Evidence suggests that choroid is thinner in myopes as compared to nonmyopes. However, choroidal thickness varies with the refractive error, age, axial length, and ethnicity. The purpose of this study was to determine the subfoveal choroidal thickness (SFCT) in high myopic Nepalese subjects and to investigate its association with the mean spherical equivalent refractive error (MSE), axial length, and age.

**Methods:**

Ninety-two eyes of 92 high myopic subjects (MSE ≤ −6 diopters) and 83 eyes of 83 emmetropic subjects (MSE: 0.00 Diopters) were included in the study. SFCT was assessed using spectral domain optical coherence tomography, and the axial length was measured using partial coherence interferometry. SFCT was measured manually using the inbuilt tool within the imaging software.

**Results:**

SFCT in the high myopic subjects was significantly thinner (mean ± SD: 224.17 ± 68.91 *μ*m) as compared to the emmetropic subjects (353.24 ± 65.63 *μ*m) (mean difference, 127.76 ± 130.80 *μ*m, and *p* < 0.001). In high myopic subjects, there was a significant negative correlation of choroidal thickness with the axial length (rho = −0.75; *p* < 0.001) and MSE (rho = −0.404; *p* < 0.01). Regression analysis demonstrated a decrease of choroidal thickness by 40.32 *μ*m (*p* < 0.001) for every 1 millimeter increase in the axial length and by 11.65 *μ*m (*p* < 0.001) for every 1 diopter increase in the MSE.

**Conclusion:**

High myopic Nepalese subjects had significantly thinner choroid as compared to emmetropes. The MSE and axial length were inversely correlated with the SFCT. Age had no effect on SFCT in this study. These findings may have implications in interpreting choroidal thickness values in clinical and epidemiological studies in myopes, especially in the south Asian population.

## 1. Introduction

The global prevalence of myopia has increased so rapidly in the recent decades that it has now become a significant public health concern [1], from East Asia [2] to the United States in the west [3]. This dramatic increase in myopia prevalence has led to an increased occurrence of several sight-threatening ocular pathologies [4]. Myopia, and particularly high myopia, is often associated with sight threatening conditions such as myopic macular degeneration, choroidal neovascularization, and retinal detachment [4, 5]. Researchers around the world have been applying untiring efforts to unravel the mysteries of myopia via epidemiological, clinical, and basic science studies. One of such efforts is in the area of understanding the role of choroid in myopia development and progression [6]. Choroid is a vascular layer between the retina and the sclera, supplying oxygen and nutrients to the outer retinal layers and can be imaged noninvasively using optical coherence tomography (OCT) [7, 8]. Studies on choroidal thickness in relation to myopia have demonstrated the role of choroid in the etiopathology of myopia, wherein the severity of myopia is directly related to the thinning of the choroid [9–11]. Choroid plays a critical role in the eyeball growth regulation [12] and that choroidal thickness responds to the manipulation of visual defocus and blur, i.e., induced myopic defocus by the use of plus lenses leads to increased choroidal thickness and induced hyperopic defocus by the use of minus lens leads to choroidal thinning [13, 14]. The change, however, appears to be temporary. The recent studies suggest that the thinning of choroidal layer can also be used as a predictor of future myopia progression and myopia related complications [15]. Therefore, the measurement of choroidal thickness is an important parameter in the assessment and management of high myopia. However, the impact of genetics and ethnic/racial differences on choroidal thickness measurement remains largely unexplored posing a significant challenge in providing personalized and effective care to patients from diverse ethnic backgrounds.

Despite the measurement of choroidal thickness (CT) being a topic of interest in the scientific community, particularly in relation to myopia progression and myopia control, there is still no consensus on the influence of genetics [16] and ethnic/racial differences on CT measurement. Studies have shown significant differences in CT among individuals from different ethnic/racial origin. In particular, a multiethnic study conducted among Asian populations, including Malay, Chinese, and Indian individuals, has identified significant differences in choroidal thickness among different ethnic groups, even after adjusting for factors such as age, gender, and axial length [17]. Additionally, a study found that the choroid is thinner in the para and perifoveal regions of Asian children compared to European children [18]. This suggests that there may be underlying genetic factors contributing to the observed differences in choroidal thickness.

Furthermore, recent research has suggested that CT measurement is influenced by the density of choroidal and retinal pigment epithelium (RPE) pigmentation [19], especially for the subfoveal region, due to signal interference caused by the retinal pigment epithelium and choroidal blood vessels. This signal interference may compromise the accurate visualization of the chorioscleral junction which might lead to over or under estimation of SFCT measurement. Macular pigmentation is relatively lower in Caucasians and higher in nonCaucasians, such as South Asians [20], including Nepalese individuals.

Given the ethnic/racial and genetic attributes which impact CT, it is imperative to measure the CT data in particular myopic racial groups to account for racial differences and use CT data specific to the group in myopia control. As such, it is essential to measure CT in high myopic subjects from unique racial groups such as Nepalese myopic subjects, where these attributes are specific to them. Data on CT from other ethnic groups may not be relevant to study CT-related changes in myopia in Nepalese subjects.

Several cross-sectional studies on subfoveal choroidal thickness (SFCT) in myopic and nonmyopic subjects are available in the literature [9, 21, 22]. These studies corroborate the findings that choroid is thinner in myopic subjects compared to age and gender matched emmetropes. Although the prevalence of myopia is higher in Asia, compared to the rest of the world, there is a lack of CT data in South-East Asian myopic subjects, particularly from Nepal. Therefore, in this study, the SFCT was assessed in high myopic Nepalese subjects and compared with age and gender-matched emmetropic subjects. Furthermore, the relationship between the axial length and the refractive error with SFCT in high myopic subjects was investigated. To the best of our knowledge, this study is the first of its kind to be conducted among Nepalese high myopic population.

## 2. Methods

A cross-sectional, case-control study was conducted at B. P. Koirala Lions Center for Ophthalmic Studies (BPKLCOS), a tertiary eye hospital in Kathmandu, Nepal, for a period of 1 year. The study was approved by the Institutional Review Committee of the Institute of Medicine, Tribhuvan University, and adhered to the Declaration of Helsinki. Written informed consent was obtained from all the participants prior to the study, which was recruited from retina and refraction OPD (out-patient department) of BPKLCOS. A detailed medical and ophthalmic history including systemic disease, ocular trauma, and ocular surgery was taken. Ninety-two healthy high myopic subjects with no systemic and ocular disease (for instance, uncontrolled diabetes or hypertension, cataract surgery, retinal surgery, and treatment) were included. Inclusion criteria for high myopic subjects were the mean spherical equivalent refractive error of ≤−6 diopters with the best corrected visual acuity of 6/9 or better. Furthermore, the subjects with ocular conditions such as macular hole, epiretinal membrane, vitreomacular traction syndrome, foveoschisis, uveitis, angioid streaks, central serous chorioretinopathy, choroidal neovascularization, keratoconus, glaucoma, and amblyopia that might alter choroidal thickness were excluded from the study. Eighty-three age and gender matched emmetropes were enrolled as control subjects. Routine ophthalmic examinations including visual acuity, objective/subjective refraction, and dilated fundus evaluation were conducted in all the participants. Both groups had normal slit-lamp biomicroscopy and funduscopy and well-defined chorioscleral junction in the OCT scan. Control subjects had a refractive error of 0.00 diopters with the visual acuity of 6/6 or better. The refractive status of the subjects was determined using dry retinoscopy as cycloplegic drugs are shown to affect choroidal thickness [23, 24].

### 2.1. Biometry

The axial length of high myopic subjects was measured using noncontact partial coherence interferometry (IOL Master 500, software version 5.00, Carl Zeiss Meditec AG, Germany). For each eye, three consecutive measurements were obtained, and the average value was taken for further calculation.

### 2.2. Optical Coherence Tomography

**Figure 1:**
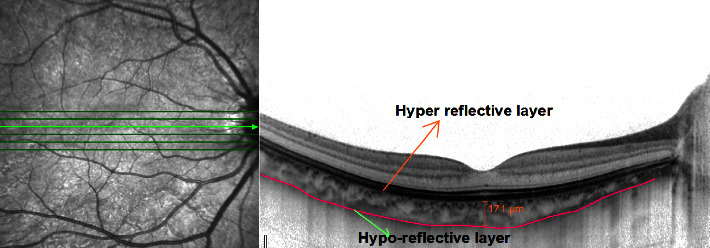
Enhanced depth imaging optical coherence tomography scan of a high myopic 24 -year-old male showing choroidal thickness with RPE-Bruch's membrane junction (the hyper-reflective layer) and the chorioscleral junction (the hyporeflective layer).

Choroidal thickness was assessed using spectral domain optical coherence tomography (Spectralis, software version 6.0, Heidelberg Engineering, Germany). The enhanced depth imaging mode, which allows for a better visualization of posterior structures such as choroid and sclera was used. Volume scans consisting of 7 lines within 30° × 5° rectangle, centered on the fovea were captured from both eyes. For optimal image quality and noise reduction, averaging was kept to 100 frames per image. The horizontal section passing directly through the center of fovea was chosen for choroidal thickness measurement. SFCT in the captured enhanced image was determined as the perpendicular distance between the outer portion of hyper-reflective layer corresponding to the retinal pigment epithelium (automatically detected by the instrument) and the hyporeflective layer corresponding to the chorioscleral junction (Figure 1). The measurement of SFCT was done by skilled optometric technician who was unaware of this study as well as the ocular history of both emmetropic and high myopic subjects. The refractive error of the subjects was adjusted by one of the authors in OCT instrument such that the measurement of SFCT remains unbiased.

All images were obtained between 9 am and 12 noon to avoid possible effects of diurnal variations in choroidal thickness [25]. For each subject, only right eye was used for further analysis.

### 2.3. Statistical Analysis

Statistical analyses were performed using IBM SPSS statistics version 21.0. (IBM Co., Armonk, NY, USA). The data were tested for normality using the Kolmogorov–Smirnov test. Normally distributed data are expressed as mean ± standard deviation (SD), and non-normal data are expressed as median and interquartile range (IQR). Spearman's correlation test and regression analysis were used to observe the association of SFCT with the axial length and the MSE. The independent t-test was used to compare SFCT between high myopic and emmetropic subjects. The *p* value <0.05 was considered statistically significant.

## 3. Results

**Figure 2:**
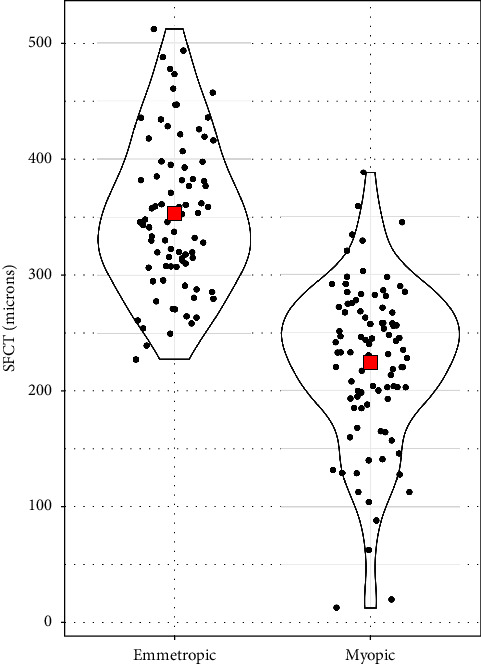
Distribution of SFCT among myopic (high myopic) and emmetropic subjects. Red diamonds denote mean values.

**Figure 3:**
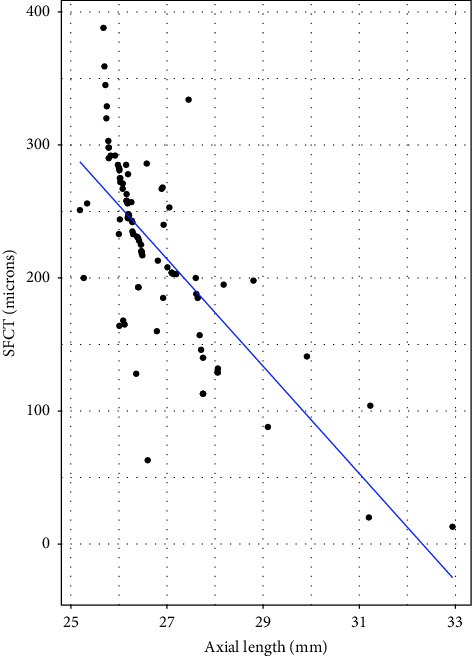
Scatterplot showing the association between the axial length and SFCT in myopic subjects.

**Figure 4:**
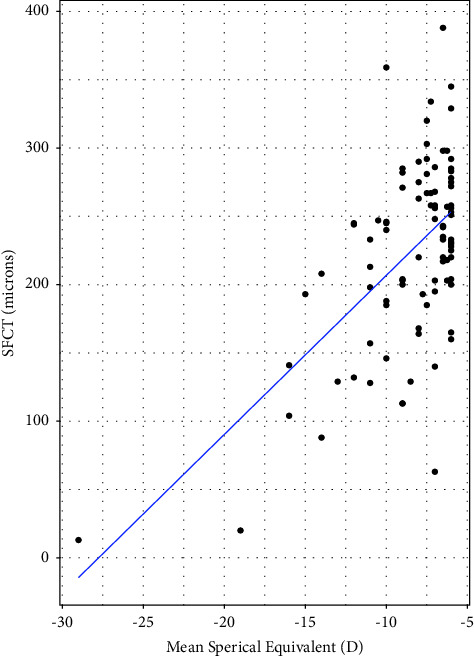
Scatter plot demonstrating the association between SFCT and the refractive error in myopic subjects.

The median age of both high myopic and emmetropic subjects was 23 years (IQR = 8 years). The median refractive error was −7.38 D (IQR = 3.75D) for high myopic subjects and 0.00 D for emmetropic subjects. The median axial length of high myopic subjects was 26.33 mm (IQR = 1.07 mm) (Table 1). The mean SFCT was 224.17 ± 68.91 *μ*m and 353.24 ± 65.63 *μ*m in high myopic and emmetropic subjects, respectively (Figure 2). High myopic subjects exhibited a significantly thinner SFCT than emmetropic subjects (mean difference ± SD = 127.76 ± 130.80 *μ*m, 95% CI: 108.93 *μ*m to 149.20 *μ*m, and *p* < 0.001). Regression analysis showed a decrease in choroidal thickness by 40.32 *μ*m (95% CI: 32.58 *μ*m to 48.06 *μ*m, and *p* < 0.001) for every 1 mm increase in the axial length. Similarly, for every 1 diopter increase in the myopic refractive error, choroidal thickness decreased by 11.65 *μ*m (95% CI: 8.24 *μ*m to 15.06 *μ*m, and *p* < 0.001) (Table 2). In high myopic subjects, both the axial length and the refractive error showed significant negative correlation with choroidal thickness (*p*=−0.75; *p* < 0.001 for the axial length (Figure 3) and *p*=−0.404; *p* < 0.01, for refractive error (Figure 4)). There was no significant association between age and SFCT in both high myopic (*p*=−0.183, *p*=0.081) and emmetropic subjects (*p*=0.123, *p*=0.270). In high myopic subjects, mean SFCT in males and females were 230.40 ± 60 *μ*m and 217.95 ± 77 *μ*m, respectively. Similarly, in emmetropic subjects, mean SFCT in males and females were 348.83 ± 66.54 *μ*m and 357.97 ± 65 *μ*m, respectively. There was no significant difference in SFCT between males and females in both high myopic subjects (mean difference: 12.43 ± 138 *μ*m, 95% CI: 16.15 *μ*m to 41.02 *μ*m, and *p*=0.390) and emmetropic subjects (mean difference: 9.13 ± 131.8 *μ*m, 95% CI: 19 *μ*m to 37.93 *μ*m, and *p*=0.530).

## 4. Discussion

In the two extant studies examining choroidal thickness in healthy Nepalese population [26, 27], distinctions in choroidal thickness measurements were observed in Nepalese subjects relative to populations of varying ethnic/racial backgrounds. Therefore, in this study, we evaluated SFCT measurements in high myopic Nepalese subjects and compared it with healthy controls to explore if similar changes in SFCT were evident in high myopic Nepalese subjects as well. We evaluated SFCT in high myopic and emmetropic Nepalese subjects using the enhanced depth imaging-spectral domain optical coherence tomography. A significant decrease in SFCT with increasing axial length and increasing myopia severity was observed. Furthermore, high myopic subjects demonstrated significantly thinner choroid compared to age- and gender-matched emmetropic subjects.

The findings show that SFCT and the axial length are inversely correlated. SFCT decreased by 40.32 *μ*m for every millimeter increase in the axial length. Similar negative correlation between the choroidal thickness and axial length has been reported by several previous studies [28–32]. Xiong et al. showed a decrease in SFCT by 18.46 *μ*m for every 1 mm increase in the axial length [33]. Gupta et al. reported decrease of SFCT by 32.31 *μ*m for every 1 mm increase in the axial length [11]. Such rate of thinning of SFCT per millimeter increase in the axial length is found to be different in different countries (Table 3), which might have been caused due to ethnic/racial differences. In our study, we found a decrease in the choroidal thickness by 11.53 *μ*m for every 1 diopter increase in the refractive error. This rate of choroidal thinning per diopter falls within the range reported in the previous studies (5.00–25.00 *μ*m) [34]. A study by Fujiwara et al. reported that SFCT decreased by 8.70 *μ*m for each diopter increase in myopia [9]. In another study, Ho et al. reported that SFCT decreased by 6.20 *μ*m for each diopter of myopia [10].

There are significant variations in SFCT measurements across studies. A few such studies are summarized in Table 3. Our values of SFCT in young adults are similar to those reported by Gupta et al. [11] but different to Kim et al. [39], Flores-Moreno et al. [22], and several others (Table 3) [9, 10, 30, 40–42]. The similarity in the choroidal thickness between the findings of Gupta et al. and our study could be due to similarities in ethnicity (Asian population) and the age range. However, the differences with Flores-Moreno et al. [22] and Kim et al. [39] studies could be due to differences in participants' characteristics such as age, axial length, refractive error, and ethnicity. The mean age and the axial length of our study population was 24 ± 7 years and 26.78 ± 1.26 mm, respectively. These values were considerably different in the studies by Flores-Moreno et al. (54 ± 18.2 years, and 29.17 ± 2.44 mm) [22] and Kim et al. [39] (42.95 ± 12.3 years and 27.28 ± 0.96 mm) [27].

High myopic subjects had, on average, 127 *μ*m thinner choroid than age-matched emmetropes in our study. This finding is in line with the previous studies that used EDI OCT imaging for measuring choroidal thickness in high myopes (see Table 3). Duan et al. assessed macular choroidal thickness in young Chinese students and found it to be thinner in high myopic eyes than emmetropic eyes (194.0 ± 59.7 *μ*m vs. 300.3 ± 62.1 *μ*m) [40]. Similarly, Heirani et al. measured SFCT in Iranian population and reported significantly thinner mean SFCT in myopic subjects compared to emmetropic subjects [42]. Wang et al. measured SFCT in 301 eyes of 171 high myopic subjects and 165 eyes of 103 normal subjects and found that the high myopic subjects had an SFCT of (200.54 ± 69.39) *μ*m, which was significantly thinner than that of the control subjects (276.21 ± 64.67) *μ*m [6]. Gupta et al. evaluated SFCT in young Chinese males in Singapore and found SFCT in high myopic and emmetropic subjects of 225.87 ± 5.51 *μ*m and 375.15 ± 6.58 *μ*m, respectively, which are very similar to the findings in our study [11]. The SFCT of emmetropic subjects reported in this study is thicker than that reported in a previous study conducted by Gyawali et al. (353.24 ± 65.63 *μ*m vs. 310.31 ± 75.70 *μ*m), which is likely due to the inclusion of relatively older patients, age ranging from 17 to 79 years [27].

These findings suggest a significant thinning of SFCT in axially elongated eyes. It is speculated that the thinning of choroid with increasing axial length could be a result of progressive degenerative changes associated with myopia and peripapillary atrophy, and increased mechanical stretching of the sclera [30, 43, 44], although recent research suggest that increase in the axial length and resultant passive stretching of sclera might not fully account for the total thinning of choroid observed in myopes.

This study did not find any association between age and choroidal thickness, contrasting with several previous studies where age has been found to be a significant predictor of SFCT [34, 44–47]. One of the reasons for this could be due to the limited spread of age of the participants enrolled in this study. Over 80% of participants were between 15 and 30 years of age.

A few limitations in this study must be acknowledged. First, the choroidal thickness was measured at a subfoveal location only. A complete choroidal thickness profile at various eccentricities of retina could have provided a more detailed understanding of the role of choroid in myopia. However, we were limited by the availability of resources (time and instrument) to capture such data. Nevertheless, choroidal thickness is most commonly studied at the subfoveal regions in myopic studies, and it has been shown that the effects on choroid due to axial elongation are more pronounced in the subfoveal region [6, 42, 39]. Second, we used the manual visual inspection method to determine SFCT. The use of advanced automated techniques could have provided more precise and accurate measurements; nevertheless, we tried to minimize the measurement bias by utilizing a single experienced observer who was masked to the subjects' refractive status during image acquisition. Moreover, we have already published choroidal thickness data form a normative population using the same method [27]. This assures the validity of the method. Third, we used noncycloplegic refraction to determine the refractive status of the enrolled subjects which is a commonly used technique in choroidal thickness studies [11]. This could have led to some discrepancies in the relationship between the refractive error and the axial length or the refractive error and SFCT between our study and others. Finally, the absolute values of SFCT measured in this study might have been influenced by the level of retinal and choroidal pigmentation [19]. Therefore, further studies must be conducted in Nepalese population to establish test-retest reliability of SFCT measurement.

## 5. Conclusion

High myopic Nepalese subjects had significantly thinner choroid compared to age- and gender-matched emmetropic subjects. The axial length and refractive error were inversely correlated with SFCT, with axial length being the greatest predictor of SFCT. The findings of this study support and corroborate the existing literature regarding the relationship between AL, MSE, and SFCT. The study provided new data on SFCT of myopic eyes from a South Asian country (Nepal). Given the fact that there is a considerable ethnic and regional variability in choroidal thickness, these new data can provide important insights into the pathophysiology of myopia in the south Asian population and aid in the development of tailored myopia management strategies.

## Figures and Tables

**Table 1:** Descriptive statistics of emmetropic and myopic groups.

Observed variables	Central tendencies	Min	Max
*Emmetropic subjects (n* *=* *83)*			
Age^Ɨ^ (years)	23	14	43
SFCT^*∗*^ (*μ*m)	353.24 ± 65.63	228.00	512.00

*Myopic subjects (n* *=* *92)*			
Age^Ɨ^ (years)	23	14	43
SFCT^*∗*^ (*μ*m)	224.17 ± 68.91	13	388
Axial length^Ɨ^ (mm)	26.33 ± 1.07	25.19	32.94
MSE^Ɨ^ (D)	−7.38 ± 3.75	−6	−29

^*∗*^Data with normal distribution reported as mean ± SD. ^Ɨ^Data with non-normal distribution reported as median ± IQR.

**Table 2:** Simple linear regression results of the predicted SFCT based on the predictor axial length and the MSE.

Predicted (dependent variables)	Predictor (independent variables)	*B*	Standard error *B*	Beta	*T*	*p*	95% confidence intervals	Regression results
SFCT (*μ*m)	Axial length (mm)	−40.32	3.90	−0.737	−10.35	<0.001	−48.06 to −32.58	*R* = 0.74
								*R* ^2^ = 0.54
								*F* = 107.18 *p*=<0.001
	MSE (D)	11.65	1.72	0.58	6.78	<0.001	8.24 to 15.06	*R* = 0.58
								*R* ^2^ = 0.39
								*F* = 46.02 *p*=<0.001

**Table 3:** Subfoveal choroidal thickness (SFCT) in high myopic population as reported in different studies.

Studies	Countries	*N*(high myopes)	Age ranges	AXL mean, range	Myopia range, MSE	EDI (yes/no)	Choroidal thickness (SFCT, *μ*m)	SFCT (*μ*m) change per 1 mm increase in axl (mm)
Flores-Moreno et al. [22]	Spain	83	18–99 years	29.17 ± 2.44 mm (26 mm to 35.63 mm)	−6 to −26 D RE: −14.3 ± 5.4 D	Yes	115.5 ± 85.3 *μ*m	25.9 ± 2.1 *μ*m

Gupta et al. [11]	Singapore	520	17–29 years	27.32 ± 1.16 mm, (no range info)	−6 to −23 D RE: −8.68 ± 2.05 D	Yes	225.87 ± 5.51 *μ*m	32.31 *μ*m

Fujiwara et al. [9]	USA	31	24–90 years	No AXL info	RE: −11.9 ± 3.7 D	Yes	93.2 ± 62.5 *μ*m	NA

Ho et al. [10]	Hongkong	56	42–62 years	No AXL info	−6.1 to −11 D (MSE: −8.7 D)	Yes	118 *μ*m ± 68 *μ*m	NA

Liu et al., [35]	China	312	18–88 years	29.45 ± 2.31 (24.31 to 36.23 mm)	−6 to −32 D −14.58 ± 5.52 D	Yes	83.77 ± 54.64 *μ*m	16.646 *μ*m

Teberik and Kaya [36]	Turkey	30	13–66 years	27.5 ± 1.2 mm	−9.6 ± 2.92 D	Yes	218.3 ± 102.25 *μ*m	34.87 *μ*m

El-Shazly et al., [37]	Egypt	120	26.07 ± 3.98 years	26.6 ± 0.59 mm	−8.31 ± 2.13 D	Yes	259.30 ± 50.92 *μ*m	33.18 *μ*m
	High myopia	60	24.45 ± 6.02 years	27.6 ± 0.61	−18.50 ± 1.86 D		172.75 ± 46.41 *μ*m	
	Advanced myopia	60						

Fledelius et al., [38]	Denmark	45	66 years	27.25 ± 2.01 mm	−8.9 ± 4.49 D	Yes	143 ± 89 *μ*m	32.29 *μ*m

Current study	Nepal	92	14–43 years	26.33 mm (25.19 mm to 32.94 mm)	(−6 D to −29 D) MSE: −7.38 D	Yes	224.17 ± 68.91 *μ*m	40.32 *μ*m

## Data Availability

The data used to support the findings of this study are available from the corresponding author upon request.
